# Nomogram for Predicting Facial Nerve Outcomes After Surgical Resection of Vestibular Schwannoma

**DOI:** 10.3389/fneur.2021.817071

**Published:** 2022-02-08

**Authors:** Yang Sun, Jianhua Yang, Tang Li, Kaiming Gao, Xiaoguang Tong

**Affiliations:** ^1^Clinical College of Neurology, Neurosurgery and Neurorehabilitation, Tianjin Medical University, Tianjin, China; ^2^Department of Neurosurgery, Tianjin Huanhu Hospital, Tianjin, China

**Keywords:** facial nerve outcomes, vestibular schwannoma, surgical, nomogram, cerebrospinal fluid fissure sign

## Abstract

**Objective:**

The facial nerve (FN) outcomes after vestibular schwannoma surgery seriously affect the social psychology and quality of life of patients. More and more attention has been paid to the protection of FN function. This study aimed to identify significant prognostic factors for FN outcomes after vestibular schwannoma surgery and create a new nomogram for predicting the rates of poor FN outcomes.

**Methods:**

Data from patients who had undergone operations for vestibular schwannoma between 2015 and 2020 were retrieved retrospectively and patients were divided into good and poor FN outcomes groups according to postoperative nerve function. The nomogram for predicting the risk of poor FN outcomes was constructed from the results of the univariate logistic regression analysis and the multivariate logistic regression analysis of the influencing factors for FN outcomes after surgical resection of vestibular schwannoma.

**Results:**

A total of 392 participants were enrolled. The univariate logistic regression analysis revealed that age, tumor size, cystic features of tumors, cerebrospinal fluid (CSF) cleft sign, tumor adhesion to the nerve, learning curve, and FN position were statistically significant. The multivariate logistic regression analysis showed that age, tumor size, cystic features of tumors, CSF cleft sign, tumor adhesion to the nerve, learning curve, and FN position were independent factors. The nomogram model was constructed according to these indicators. At the last follow-up examination, a good FN outcome was observed in 342 patients (87.2%) and only 50 patients (12.8%) was presented with poor FN function. Application of the nomogram in the validation cohort still gave good discrimination [area under the curve (AUC), 0.806 (95% CI, 0.752–0.861)] and good calibration.

**Conclusion:**

This study has presented a reliable and valuable nomogram that can accurately predict the occurrence of poor FN outcomes after surgery in patients. This tool is easy to use and could assist doctors in establishing clinical decision-making for individual patients.

## Introduction

Vestibular schwannoma (VS) is the most common tumor in the cerebellopontine angle (CPA) and constitutes about 6% of all the intracranial tumors (1). Since VS is histologically benign, a small asymptomatic tumor is usually treated with conservative strategy by “watchful waiting” (2, 3). But, surgery is still recommended for symptomatic and growing tumors (4). Because the operation of VS is not usually life-threatening, effective prevention of facial nerve (FN) function is critical in clinical practice.

As we all know, many factors have been associated with FN outcomes after surgery. It has been suggested that age, tumor size, and FN position all can impact ultimate facial functional outcomes (5, 6). However, other risk factors affecting FN function remain controversial (6). For instance, some authors reported cystic features of the tumors association with worse outcomes (7), whereas others showed that there is no difference between cystic and solid tumors in terms of FN function after surgery (8). To the best of our knowledge, no established method allows precise prediction of FN outcomes (9).

As a MRI feature, cerebrospinal fluid (CSF) fissure sign was often used to determining whether an intracranial mass is intra-axial or extra-axial (10). It has been reported that the CSF sign in the image of VS can evaluate the degree of adhesion between the tumor and surrounding tissues (11). As one of the innovations of this article, we confirmed that CSF cleft sign is an independent risk factor for the prognosis of FN and incorporated it into the clinical predictive model.

Based on these predictors, we hope to construct a reliable and valuable nomogram for predicting FN outcomes after surgical resection of VS. As far as we know, no nomogram is available for predicting FN outcomes after surgery.

## Materials and Methods

### Patients

A total of 392 consecutive patients who underwent surgery for VS at the Neurosurgery Department of the Tianjin Huanhu Hospital between January 1, 2015 and July 30, 2020 were retrospectively enrolled (Table 1). The Ethics Committee of our institution approved this study with a waiver of informed consent before its initiation. All the procedures were performed in accordance with the Code of Ethics of the World Medical Association (Declaration of Helsinki). The patients enrolled in this study underwent surgery via the retrosigmoid approach for VS. The exclusion criteria included previous VS microsurgical resection or radiation, resection by techniques other than the retrosigmoid approach, patients with neurofibromatosis type 2, and preoperative FN dysfunction.

**Table 1:** Population study characteristics.

**Characteristics**	**Value**
Male/Female, *N* (%)	157(40.1%)/235(59. 9%)
Age (years)	55.33 ± 12.78
Hypertension, *N* (%)	218(55.6%)
Diabetes mellitus, *N* (%)	55(14%)
Active smoker, *N* (%)	113(28.8%)
Frequent alcohol consumption, *N* (%)	158(40.3%)
Preoperative symptoms and signs *N* (%)
Trigeminal hypoesthesia	170(43.4%)
Imbalance	152(38.8%)
Tinnitus	130(33.2%)
Vertigo	54(13.8%)
Cerebellar ataxia	48(12.2%)
Intermedius nerve disturbances	32(8.2%)
Long tract signs	8(2%)
Trigeminal neuralgia	4(1%)
Follow-up (months)	38 ± 12.2

*Data presented as mean±standard deviation or N (%)*.

### Surgery and Clinical Examination

**Figure 1:**
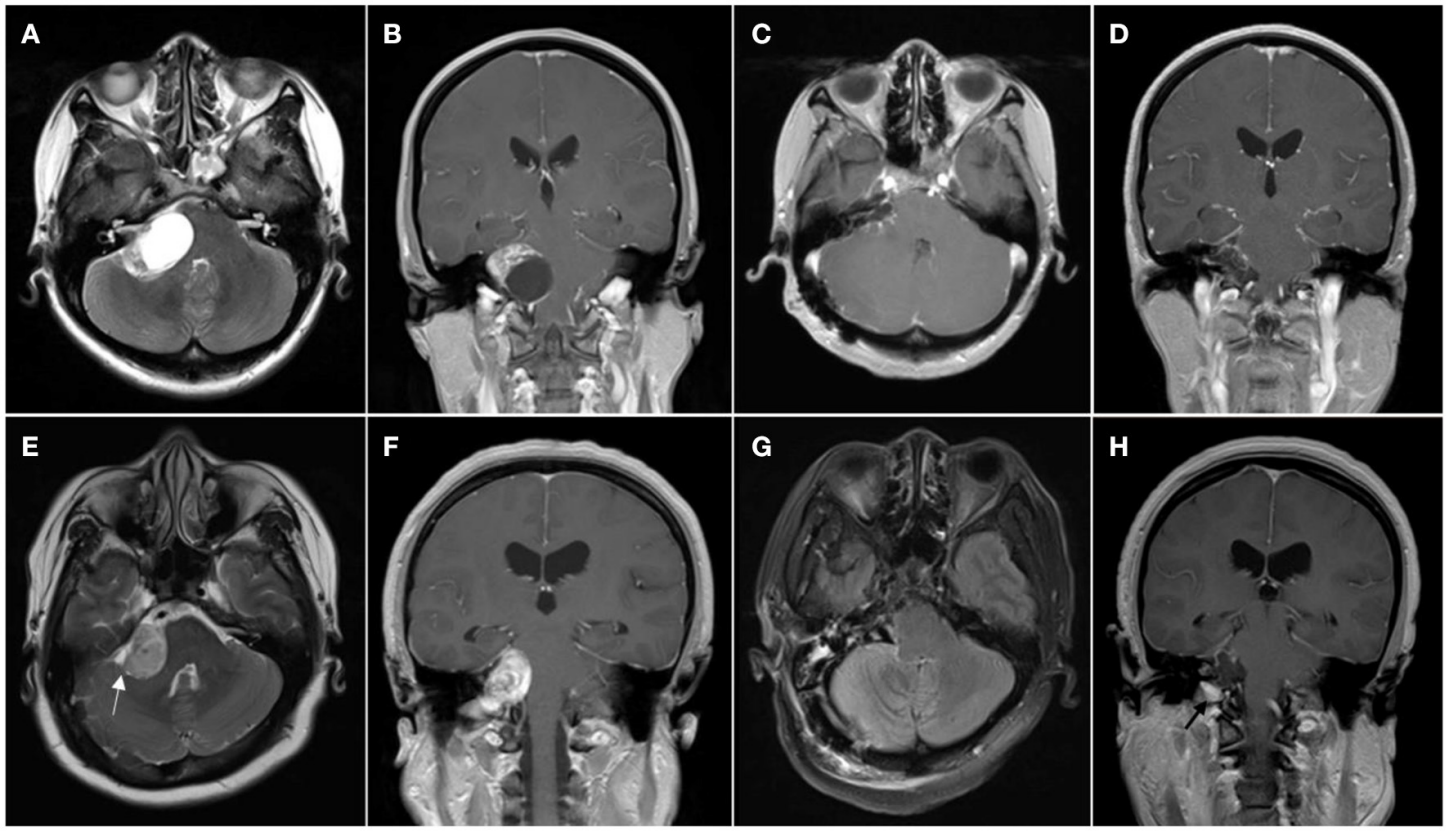
Patient with cystic vestibular schwannoma. **(A,B)** Preoperative MR images showing cystic vestibular schwannoma; **(C,D)** Postoperative MR images showing resection of the tumor. **(E–H)** Patient with solid vestibular schwannoma; **(E,F)** Preoperative MR images showing solid vestibular schwannoma, cerebrospinal fluid (CSF) cleft sign surrounding the tumor is marked with the white arrowhead; and **(G,H)** Postoperative MR images showing resection of the tumor and left tumor remnant is marked with the black arrowhead.

All the cases in this study were performed by the same team of surgeons. All the patients were operated by the retrosigmoid approach and pathologically diagnosed as VS. The FN monitoring was used during surgery. Routine preoperative examinations included pre- and postoperative head CT, pre- and postoperative head MRI, and pathological examination (Figure 1).

### Clinical Variables

The clinical characteristics of the participants are given in Table 2. The data were obtained primarily from the clinical medical records of patients and the radiology databases of our hospital. The variables included gender, age, duration of symptoms, preoperative hearing loss, tumor location, tumor size, internal auditory canal width, the extent of resection, cystic features of tumors, brainstem or cerebellar edema, displacement patterns of the FN, surgical time, CSF cleft sign, and learning curve. The outcomes of preoperative hearing status were determined according to the foundation of the American Academy of Otolaryngology-Head and Neck Surgery. The serviceable hearing was defined as a speech discrimination score (SDS) ≥ 50% and a pure tone average (PTA) ≤ 50 dB. Tumor size was measured as the largest diameter of the tumor in the CPA without considering the intracanalicular component of the lesion. The extent of resection was divided into 3 types: gross total removal (GTR) was complete excision of the tumor. Only a thin layer of tumor remained on one or more nerves was defined as near total resection (NTR). MRI revealed gross evidence of residual disease and then it was termed as subtotal resection (STR) (12). Cystic features of tumors, CSF cleft sign surrounding the tumor, and brainstem compression were identified by preoperative MRI and intraoperative observation. Displacement patterns of the FN, which were classed into 4 types (type 1: FN anterior to VS; type 2: FN anterior and inferior to VS; type 3: FN superior to VS; and type 4: FN posterior to the tumor), were confirmed by MRI and verified by intraoperative observation (13). To evaluate the improvement in procedural results due to the experience progressively acquired by the surgeon (learning curve), we transformed the FN poor outcome into the cumulative rate of FN poor outcome and determined the cutoff value of learning curve through the receiver operating characteristic (ROC) curve (Supplementary Figure S1). The patients were subdivided into the 2 different groups according to the cutoff value (number 1 through 186 and number 206 through 392). The outcomes of FN were assessed using the House–Brackmann grading system (14) and it is usually evaluated 5–7 days after operation. The House–Brackmann grade I or II facial functions was categorized as good FN outcomes and the House–Brackmann grades III to VI facial functions were categorized as poor FN outcomes.

**Table 2:** The univariate logistic regression analysis of facial nerve outcomes after surgical resection of vestibular schwannoma.

	**Statistics**	**OR, 95% CI**	***P*-value**
**Gender**			0.923
Female	235(59.9%)	1	
Male	157(40.1%)	1.021 (0.636, 1.638)	
**Age(years)**	55.33 ± 12.78	1.020 (1.000, 1.039)	0.047^*^
**Duration of symptoms (months)**	11.66 ± 4.52	1.004 (0.954, 1.057)	0.872
**Preoperative hearing status**			0.432
Unserviceable hearing	203 (51.8%)	1	
Serviceable hearing	189 (48.2%)	1.205 (0.757, 1.92)	
**Tumor location**			0.828
Left	188 (48%)	1	
Right	204 (52%)	0.950 (0.597, 1.511)	
**Tumor size (mm)**	31.64 ± 10.61	1.082 (1.056, 1.109)	<0.001^*^
**CSF cleft sign**			<0.001^*^
No	152 (38.8%)	1	
Yes	240 (61.2%)	0.210 (0.128, 0.344)	
**Cystic features of tumors**			0.004^*^
No	336 (84.9%)	1	
Yes	59 (15.1%)	2.358 (1.313, 4.233)	
**Tumor heterogeneity**			0.341
No	110 (28.1%)	1	
Yes	282 (71.9%)	1.277 (0.772, 2.114)	
**IAC width (mm)**	7.96 ± 1.53	1.060 (0.911, 1.234)	0.449
**Brainstem or cerebellar edema**			0.237
No	225 (57.4%)	1	
Yes	167 (42.6%)	1.325 (0.831, 2.111)	
**Facial nerve position**
Type1	152 (38.8%)	1	0.002^*^
Type2	186 (47.4%)	2.417 (1.392, 4.197)	0.002^*^
Type3	54 (13.8%)	2.955 (1.432, 6.095)	0.003^*^
**Extent of resection**
GTR	224 (57.1%)	1	0.395
NTR	124 (31.6%)	1.385 (0.834, 2.301)	0.208
STR	44 (11.3%)	1.375 (0.659, 2.871)	0.397
**Surgical time (min)**	306.48 ± 83.53	1.002 (0.999, 1.005)	0.133
**Learning curve**			0.004^*^
Group1 (Early stage)	186 (47.5%)	1	
Group2 (Late stage)	206 (52.5%)	0.495 (0.309, 0.795)	

*Data presented as mean ± SD or n (%) and the effect sizes were expressed as OR (95% CI), p-value.*

* *Statistically significant. OR, odds ratio*.

### Statistical Analyses

Statistical analyses were performed using the SPSS software program, version 19.0, for Windows (IBM Corporation, Armonk, New York, USA). The *t*-test, the chi-squared test, or the rank-sum test was used for single factor analysis. The significance of each variable was assessed using the univariate logistic regression analysis, which investigated the independent risk factors for FN outcomes. Additional variables significantly related to FN outcomes in the univariate models (*p* < 0.05) were subsequently included in the multivariate model. A nomogram was performed using the rms package of R, version 3.0 (http://www.r-project.org/) based on the results of the multivariate logistic regression analysis (15). The Hosmer–Lemeshow goodness-of-fit test was used to evaluate the calibration degree of the nomogram model and the ROC curve of the nomogram model was calculated. The area under the curve (AUC) of the ROC analysis was used to measure the performance of the nomogram. In all the analyses, *p* < 0.05 indicated statistical significance.

## Results

### Univariate Logistic Regression Analysis of FN Outcomes After Surgical Resection of VS

Statistically significant differences were found between the 2 groups in age, tumor size, cystic features of tumors, CSF cleft surrounding the tumor, learning curve, and FN position (*p* < 0.05; Table 2).

### Multivariate Logistic Regression Analysis of FN Outcomes After Surgical Resection of VS

The statistically significant factors were analyzed by the multivariate logistic regression analysis as the dependent variables. These included age, tumor size, cystic features of tumors, CSF cleft sign, learning curve, and FN position. Variable assignment classification was used for the categorical variables and their assignment was either no and weak, indicated by 0, or yes and strong, indicated by 1. The variables of FN position were assigned as follows: type 1, indicated by 0; type 2, indicated by 1; and type 3, indicated by 2. The measurement data included age, tumor size, cystic features of tumors, CSF cleft surrounding the tumor, learning curve, and FN position. The multivariate logistic regression analysis and variable screening using stepwise method (model inclusion level, 0.05; rejection level, 0.10). The results of the likelihood ratio chi-squared test showed that the regression model had statistical significance (*p* < 0.05) and the Hosmer–Lemeshow goodness-of-fit test showed that the model had an excellent calibration degree (*I*^2^ = 10.185; *p* = 0.252). In addition, age, tumor size, CSF cleft sign, cystic features of tumors, learning curve, and FN position were independent factors influencing FN outcomes after surgical resection of VS (Table 3).

**Table 3:** The multivariate logistic regression analysis of facial nerve outcomes after surgical resection of vestibular schwannoma.

**Variable**	**Wals**	***P-*Value**	**OR**	**95% CI**
Age	14.960	<0.001	1.047	1.023–1.071
Tumor size	43.663	<0.001	1.128	1.089–1.169
CSF cleft sign	5.813	0.016	0.375	0.169–0.832
Cystic features of tumors	8.779	0.003	2.845	1.425–5.682
Learning curve	7.911	0.005	0.450	0.258–0.785
Facial nerve position				
Type1	14.340	0.001	Ref	Ref
Type2	11.589	0.001	3.016	1.597–5.693
Type3	10.012	0.002	3.809	1.664–8.717

*OR, odds ratio; NA, not applicable; Ref, reference*.

### Nomogram of FN Outcomes After Surgical Resection of VS

**Figure 2:**
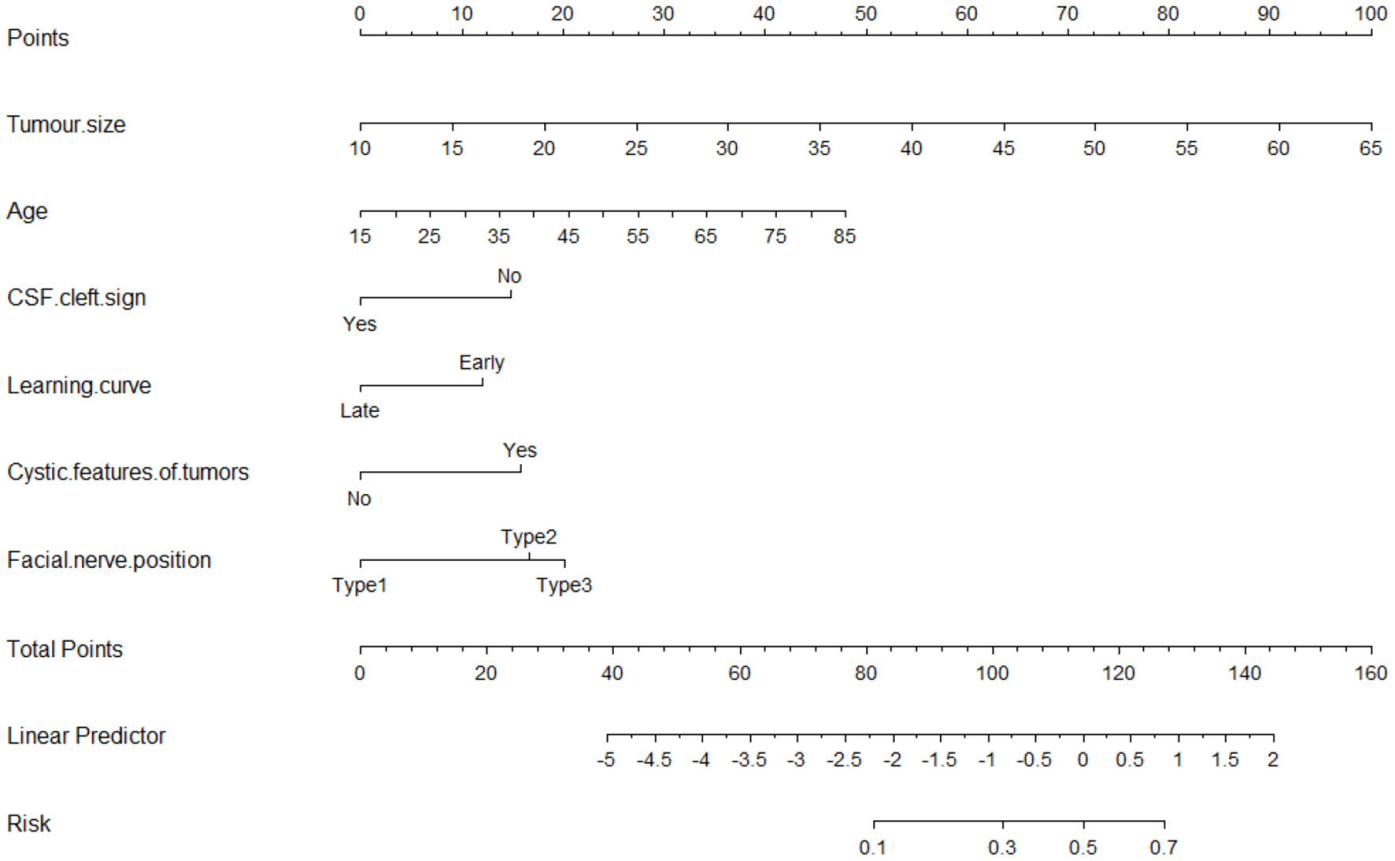
The nomogram for predicting tumor size, age, cystic features of tumors, FN position, learning curve, and CSF cleft sign to the nerve for facial nerve (FN) outcomes after surgical resection of vestibular schwannoma.

The nomogram for predicting poor FN outcomes is shown in Figure 2. The nomogram was developed using the 6 independent predictive factors (i.e., age, tumor size, CSF cleft sign, cystic features of tumors, learning curve, and FN position). Each factor in the nomogram was assigned a weighted number of points and the sum of the points for each patient was associated with a specific prediction of FN outcomes. For example, for a 45-year-old patient with VS (20 points) and a 45-mm diameter tumor (63 points), no CSF cleft sign (14 points), a late-stage operation (0 point), cystic features of tumors (15 points), and 2 types of FN position (18 points), the total score would be 130 points. For this patient, the prediction of poor FN outcomes after surgery would be 70% (Figure 2).

**Figure 3:**
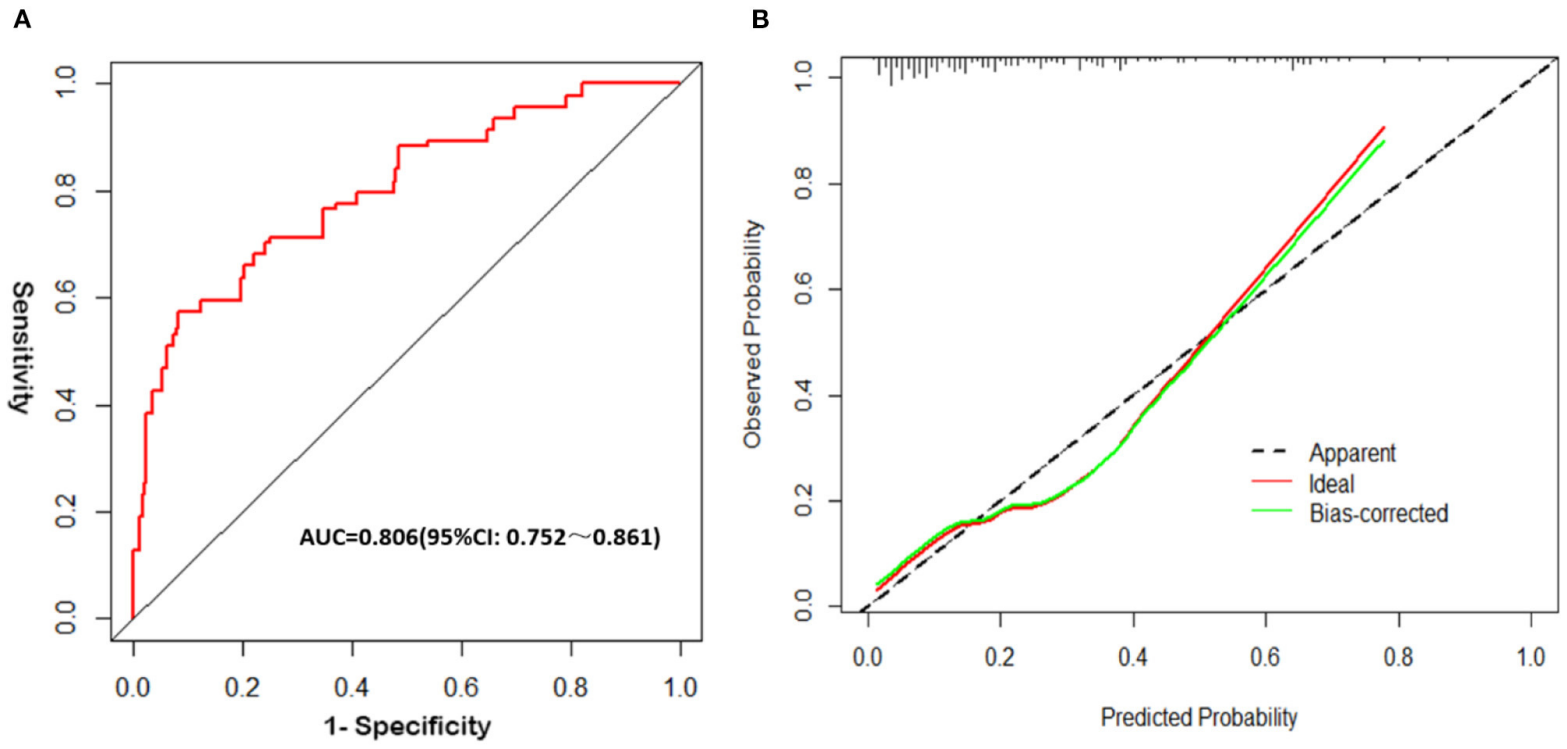
Validation of the nomogram. **(A)** The receiver operating characteristic (ROC) curve analysis of the nomogram; **(B)** Calibration curves of the nomogram.

The analysis results showed that the AUC_ROC_ was 0.806 (95% CI, 0.752–0.861; *p* < 0.001), sensitivity was 77.31%, and specificity was 85.14% (Figure 3A). Good calibration was observed for the probability of occurrence of poor FN outcomes (Figure 3B). The Hosmer–Lemeshow goodness-of-fit test yielded a nonsignificant statistic (*p* = 0.196). This nomogram can predict the risk of poor FN outcomes after surgery individually according to the distinct conditions of different patients.

### Follow-Up of FN Outcomes

**Figure 4:**
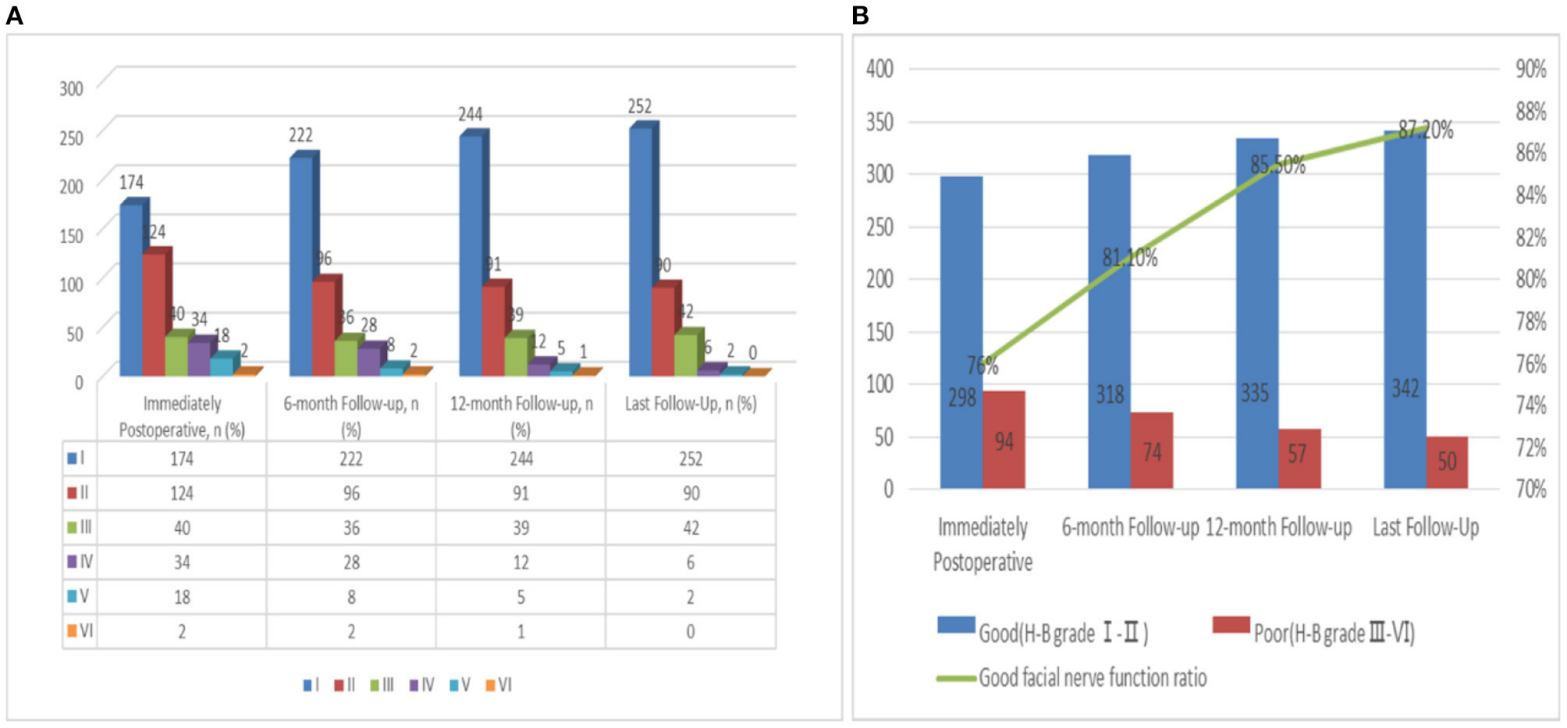
**(A)** Follow up of FN functional recovery; **(B)** Change trend of the recovery rate of FN function with follow-up time.

Tumor excision was achieved in 392 patients: 76% (*n* = 298) had a good FN outcome (H-B grades I-II) and 24% (*n* = 94) had a poor FN outcome (H-B grades III–VI) (Supplementary Table 2 and Figure 4). At the last follow-up examination, a good FN outcome was observed in 342 patients (87.2%) and only 50 patients (12.8%) presented with poor FN function (Figure 4A). With the increase of follow-up time, the neurological function of some patients recovered after the operation, but the recovery rate of FN decreased significantly after more than 1 year (Figure 4B). The median follow-up for these patients was 38 months (Table 1).

## Discussion

It is well known that the intracranial intra-arachnoidal FN is covered by a sheath of arachnoid membrane and lacks a peri- and epineural layer (16). The risk of FN injury is high in the operation of VS because of FN particular vulnerability to stretching (lack of perineurium) and reduced resistance to compression (lack of epineurium) (17). In this study, approximately 24% of patients with surgical resection of VS had poor FN outcomes (H-B grades III–VI) at discharge. The postoperative rate of poor FN outcomes decreased to 12.8% at the last follow-up. This study suggests that age, tumor size, cystic features of tumors, learning curve, and FN position are significantly associated with postoperative poor FN outcomes in the Chinese population. Considering that poor FN outcomes significantly impact the quality of life of patients, the surgeon needs to study the factors associated with the onset of FN injury (18, 19).

As we all know, many factors have been associated with FN outcomes after surgery. Among these, tumor size has been unanimously considered the most important factor in previously published clinical studies (6, 8, 9, 12, 19–22). Also, in this study, an analysis of factors affecting FN outcomes showed a highly statistically significant relationship between tumor size and FN outcomes in the univariate (*t* = 7.149, *p* < 0.001) and multivariate logistic regression analyses (*I*^2^ = 43.663, *p* < 0.001). In this study, age (*t* = 2.001, *p* = 0.046) and learning curve (*I*^2^ = 8.626, *p* = 0.004) were also significant risk factors for a poor FN outcome. The reason for a poor FN outcome in aged patients remains unclear (23). For the learning curve study, these results confirmed that the percentage of good functional FN outcomes has increased with experience (21, 24). The impact of the learning curve can be minimized in the future through professional training (21). Rhoton et al. (25) described the different relative positions of FN and acoustic neuroma and divided them into 4 types (type 1: FN anterior to VS; type 2: FN anterior and inferior to VS; type 3: FN superior to VS; and type 4: FN posterior to the tumor). Furthermore, Esquia-Medina et al. (13) reported that different FN positions would affect FN outcomes after surgical resection of VS. The correlation between FN outcomes and FN locations was confirmed again in this study (*Z* = 4.488, *p* < 0.001). The FN rarely appears on the posterior surface of the tumor (2%) (26), so all the articles including ours lack data and conclusions of type 4.

This study found that cystic features of tumors are a risk factor for predicting FN outcomes (chi-squared test = 8.779, *p* = 0.003). However, the cystic characteristics of tumors are still a controversial factor. Some authors believed that cystic features were associated with FN outcomes (27), while others had the opposite conclusion (21). The reason for controversy is that there are various descriptions of cystic features of tumors and there is no general classification system for these tumors (28). The difference in statistical conclusions is caused by the inconsistency of inclusion criteria. The grade of resection is also controversial, with some studies showing no association of grade of resection with FN outcomes (29) and with other studies showing an association with poor FN outcomes (30). There was no correlation between extent of resection and prognosis of FN in our separate analysis (*Z* = 1.139, *p* = 0.187, Supplementary Table 1). This controversy may be due to the small number of cases in this study, resulting in no significant statistical difference. Another reason is that each surgeon has a different strategy for tumor removal. The surgeon is commonly faced with balancing aggressive dissection to separate the nerve, which may result in injury, with less aggressive resection, which may risk tumor growth (30). For the tumor with strong adhesion, each surgeon chooses different, if the pursuit of total resection will increase the probability of FN injury.

As we all know, the degree of FN adhesion is also an essential factor of nerve function behind the operation. Torres et al. (19) and Veronezi et al. (31) both found that the degree of adhesion between tumor and nerve is an independent risk factor for FN outcomes after surgery in their respective studies. In this study, the establishment of the clinical nomogram model needs to predict the injury probability of FN before operation, so the degree of tumor adhesion was not included in this study. The main reason is that the degree of tumor adhesion needs to be judged by the surgeon during the operation, which cannot be judged before the operation. In addition, due to the lack of objective judgment standard, the adhesion degree depends on the subjective judgment of the surgeon and the evaluation of different doctors is easy to cause great deviation. CSF cleft sign refers to cleft sign on T2 that represents CSF spaces in the brain–tumor interface (32). Thenier-Villa et al. (33) found that negative cleft sign in the image subtraction is a predictor of postoperative neurological deficit. Yin et al. (11) reported that CSF cleft sign could be used to predict the degree of tumor–brain adhesion of VSs. The disappearance of CSF cleft sign may indicate the absence of an arachnoid plane and resulting in adhesion between a tumor and the brain, brainstem, or adjacent cranial nerves (33). CSF cleft sign can predict the degree of tumor adhesion before operation (33, 34), so we used it to replace the degree of tumor adhesion into the study of prediction model. The degree of adhesion can be determined by preoperative MRI examination. Compared with the degree of adhesion determined by the doctor during the operation, CSF fissure sign is more objective and intuitive. This study further confirmed that that the disappearance of CSF cleft sign was an independent risk factor for FN outcomes (*I*^2^ = 41.555, *p* < 0.001).

It is generally accepted that the time course of recovery in patients with postoperative impaired FN function spans the first year after surgery (35). In postoperative follow-up, we found that the earlier the recovery started, the better the quality of recovery (Figure 4A). In addition, we also observed patients with more than 1 year or even 2 years of delayed recovery, but the recovery rate of FN decreased significantly after more than 1 year (Figure 4B). For patients with anatomic preservation of the nerve, the recovery of FN function is possible. Therefore, it is essential to choose the appropriate FN reconstruction time. Combined with the results of this study, we also agree with the recommendation that reconstruction surgery should be considered if FN injury does not recover at 1 year (9, 36).

In the recent years, the nomogram has been used to construct prediction models to predict the prognosis and risk of diseases as the most commonly used method (37). The nomogram model based on the results of a multifactor analysis can make a personalized and accurate prediction on the possibility of medical events. The nomogram can be used to analyze the risk of each patient rather than summarize the risk of a group of patients. The AUC value of our nomogram was 0.806 (95% CI, 0.752–0.861), which demonstrates that the calibration of the nomogram was relatively accurate. Therefore, this nomogram has a relatively high ability to predict FN outcomes of patients.

Although the nomogram model demonstrated high accuracy for predicting FN outcomes, several limitations in our data must be considered. Specifically, the inclusion of retrospective data in this study may lead to the possibility of selection bias in the results. Moreover, all the patients in our cohort study were Asian, so the predicted results may not be accurate for patients of other races. Additionally, this study was a retrospective study and we were unable to verify our results through an external validation. This might have introduced a potential source of bias in this study. In addition, this study cannot determine more predictive factors limited by the sample size. Therefore, the later stage of this study still needs to be verified in an independent data set and further large sample and multicenter prospective research is needed.

## Conclusion

We found that age, tumor size, CSF cleft sign, cystic features of tumors, learning curve, and FN position were independent predictive factors for FN outcomes. A nomogram was constructed by combining six preoperative risk factors of FN outcomes. This model provides an efficient preoperative estimation of FN outcomes risk in patients after VS surgery. Reconstruction of FN can be recommended for patients who do not recover FN function 1 year after the operation.

## Data Availability Statement

The original contributions presented in the study are included in the article/Supplementary Material, further inquiries can be directed to the corresponding author/s.

## Ethics Statement

The studies involving human participants were reviewed and approved by Ethics Committee of Tianjin Huanhu Hospital. The patients/participants provided their written informed consent to participate in this study.

## Author Contributions

XT and YS: contributed to the conceptualization. XT, YS, JY, TL, and KG: contributed to the data curation. YS and JY: contributed to the formal analysis and methodology. XT: contributed to the project administration. YS: contributed to the writing—original draft. All authors contributed to the visualization and writing—review and editing of the final version of the manuscript.

## Funding

This study was supported by the foundation for the Science and Technology Major Project of Tianjin (Grand No. 18ZXDBSY00180) and the Tianjin Jinnan District Science and Technology Planning Project (Grand No. 20210104).

## Conflict of Interest

The authors declare that the research was conducted in the absence of any commercial or financial relationships that could be construed as a potential conflict of interest.

## Publisher's Note

All claims expressed in this article are solely those of the authors and do not necessarily represent those of their affiliated organizations, or those of the publisher, the editors and the reviewers. Any product that may be evaluated in this article, or claim that may be made by its manufacturer, is not guaranteed or endorsed by the publisher.
