# Supplementary material for: Nomogram for Predicting Facial Nerve Outcomes After Surgical Resection of Vestibular Schwannoma
Source: Front Neurol. 2022 Feb 8;12:817071. doi: 10.3389/fneur.2021.817071 (PMC8860821; doi:10.3389/fneur.2021.817071)
Supplement: Supplementary file 1 [file Table_1.docx]

**Supplementary Table 1. Demographic Characteristics of the Patients of the Study**

| **Variable** | **H-BⅠ-Ⅱ** | **H-BⅢ-Ⅵ** | **Statistic** | ***P* Value** |
| --- | --- | --- | --- | --- |
| **Gender** |  |  | ꭓ^2^=0.007 | 0.932 |
| Female | 179 | 56 |  |  |
| Male | 119 | 38 |  |  |
| **Age(years)** | 54.60±12.78 | 57.61±12.57 | *t*=2.001 | 0.046* |
| **Duration of symptoms (months)** | 11.53±4.09 | 11.72±5.68 | *t*=0.160 | 0.873 |
| **Preoperative hearing status** |  |  | ꭓ^2^=0.618 | 0.432 |
| Serviceable hearing | 147 | 42 |  |  |
| Unserviceable hearing | 151 | 52 |  |  |
| **Tumor location** |  |  | ꭓ2=0.047 | 0.828 |
| Left | 142 | 46 |  |  |
| Right | 156 | 48 |  |  |
| **Tumour size (mm)** | 29.62±9.79 | 38.06±10.59 | *t*=7.149 | <0.001* |
| **CSF cleft sign** |  |  | ꭓ^2^=41.555 | <0.001* |
| No | 89 | 63 |  |  |
| Yes | 209 | 31 |  |  |
| **Cystic features of tumors** |  |  | ꭓ^2^=8.576 | 0.003* |
| No | 262 | 71 |  |  |
| Yes | 36 | 23 |  |  |
| **Tumour heterogeneity** |  |  | ꭓ2=0.910 | 0.340 |
| No | 80 | 30 |  |  |
| Yes | 218 | 64 |  |  |
| **IAC width (mm)** | 7.92±1.32 | 8.06±1.58 | *t*=0.755 | 0.450 |
| **Brainstem or cerebellar edema** |  |  | ꭓ^2^=1.405 | 0.236 |
| No | 176 | 49 |  |  |
| Yes | 122 | 45 |  |  |
| **Facial nerve position** |  |  | *Z*=3.488 | <0.001* |
| Type1 | 130 | 22 |  |  |
| Type2 | 132 | 54 |  |  |
| Type3 | 36 | 18 |  |  |
| **Extent of resection** |  |  | *Z*=1.319 | 0.187 |
| GTR | 176 | 48 |  |  |
| NTR | 90 | 34 |  |  |
| STR | 32 | 12 |  |  |
| **Surgical time (minutes)** | 302.91±86.65 | 317.8±71,99 | *t*=1.510 | 0.132 |
| **Learning curve** |  |  | ꭓ^2^=9.460 | 0.002* |
| Group1(Early stage) | 136 | 60 |  |  |
| Group2(Late stage) | 162 | 34 |  |  |
| Data presented as mean±standard deviation or n(%)  *Statistically significant | | | | |
