# Supplementary material for: Nomogram for Predicting Facial Nerve Outcomes After Surgical Resection of Vestibular Schwannoma
Source: Front Neurol. 2022 Feb 8;12:817071. doi: 10.3389/fneur.2021.817071 (PMC8860821; doi:10.3389/fneur.2021.817071)
Supplement: Supplementary file 2 [file Table_2.docx]

**Supplementary Table 2. Postoperative facial nerve function after surgical resection of vestibular schwannoma**

| **House-Brackmann Scale** | **Immediately Postoperative, n (%)** | **6-month Follow-up, n (%)** | **12-month Follow-up, n (%)** | **Last Follow-Up, n (%)** |
| --- | --- | --- | --- | --- |
|  | Good facial nerve outcome | | | |
| I | 174(44.4) | 222(56.6) | 244(62.2) | 252(64.3) |
| II | 124(31.6) | 96(24.5) | 91(23.2) | 90(23.0) |
|  | Poor facial nerve outcome | | | |
| III | 40(10.2) | 36(9.2) | 39(9.9) | 42(10.7) |
| IV | 34(8.7) | 28(7.1) | 12(3.1) | 6(1.5) |
| V | 18(4.6) | 8(2.1) | 5(1.3) | 2(0.5) |
| VI | 2(0.5) | 2(0.5) | 1(0.3) | 0 |
