# Supplementary figures and images for: Nomogram for Predicting Facial Nerve Outcomes After Surgical Resection of Vestibular Schwannoma
Source: Front Neurol. 2022 Feb 8;12:817071. doi: 10.3389/fneur.2021.817071 (PMC8860821; doi:10.3389/fneur.2021.817071)

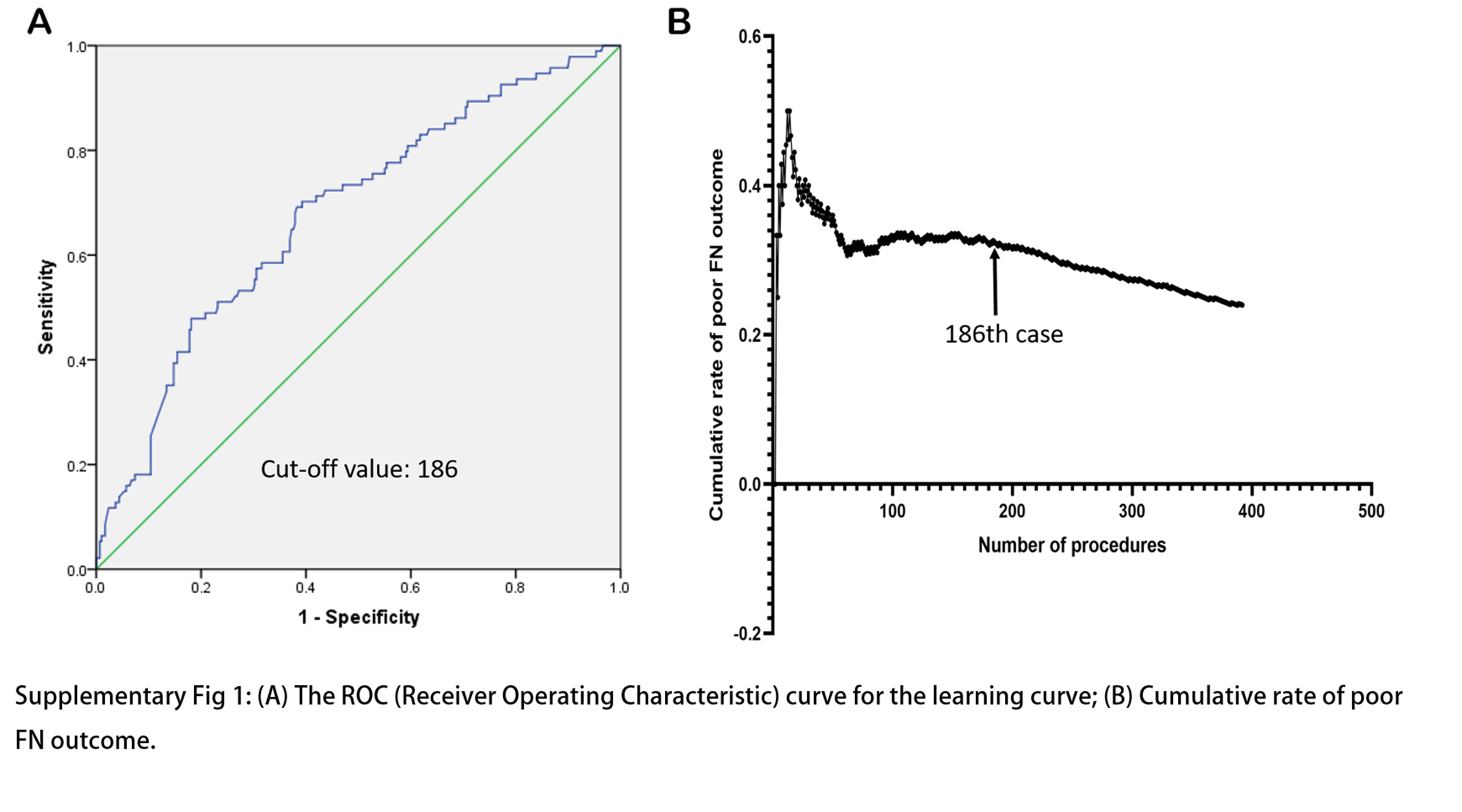

Supplement: Supplementary file 3 [file Image_1.TIF]
